# Supplementary material for: Heme Oxygenase-1 is a Key Molecule Underlying Differential Response of TW-37-Induced Apoptosis in Human Mucoepidermoid Carcinoma Cells
Source: Molecules. 2019 May 1;24(9):1700. doi: 10.3390/molecules24091700 (PMC6539960; doi:10.3390/molecules24091700)
Supplement: Supplementary file 1 [file molecules-24-01700-s001.pdf]

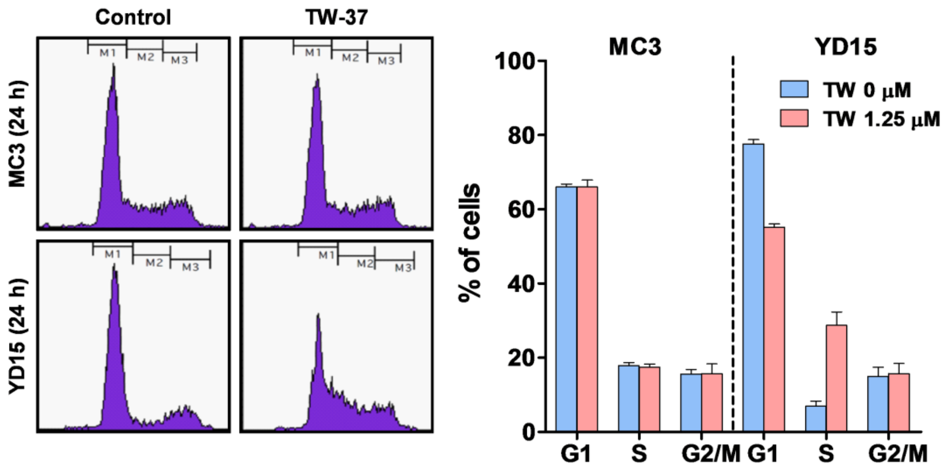

Figure S1. Effect of TW-37 on cell cycle arrest of human MEC cells. MC3 and YD-15 cells were treated with DMSO or a 1.25 μM concentration of TW-37 for 24 h. The histogram represents the distribution of the cell cycle. The results reflect three independent experiments.

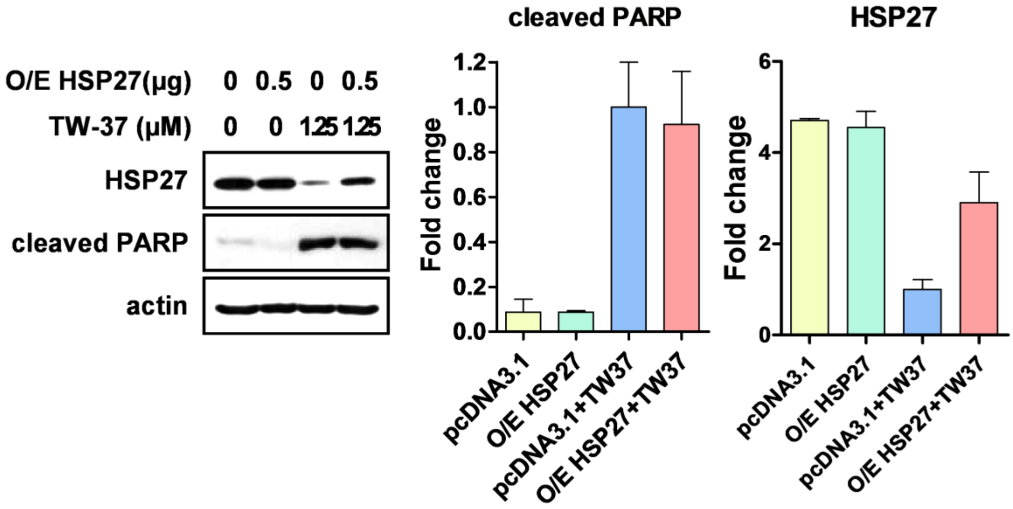

Figure S2. Effect of ectopic expression of HSP27 on TW-37-induced apoptosis. YD-15 cells were transiently transfected with pcDNA3.1 or pcDNA3.1-HSP27 for 6 h, and then treated with DMSO or TW-37 for 48 h. Protein levels of HSP27 and cleaved PARP were analyzed by western blotting. Actin was used as a loading control. Bar graphs are expressed as mean ± SD from three independent experiments.

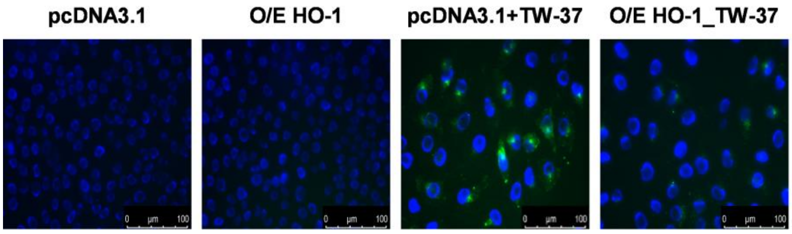

Figure S3. Effect of ectopic expression of HO-1 on TW-37-induced ROS. Intracellular levels of ROS in cells were detected using H2DCF-DA fluorescent dye and visualized by fluorescence microscope (magnification, X400).
